# Footprint measurement methods for the assessment and classification of foot types in subjects with Down syndrome: a systematic review

**DOI:** 10.1186/s13018-021-02667-0

**Published:** 2021-08-27

**Authors:** Lourdes Gutiérrez-Vilahú, Myriam Guerra-Balic

**Affiliations:** Research group on Health, Physical Activity and Sport, Faculty of Psychology, Education and Sport Sciences-Blanquerna, University Ramon Llull, C/ Císter 34, 08022 Barcelona, Spain

**Keywords:** Down syndrome, Footprint, Foot type, Clinical classification, Measurement methods, Reliability

## Abstract

**Background:**

Musculoskeletal disorders, especially in the feet, are common in people with Down syndrome (DS). Evaluation of podiatric footprints is important to prevent and manage orthopedic symptoms. The reliability of a wide variety of footprint measurement methods has been evaluated in healthy people, but few studies have considered the specific morphotype features of the feet in subjects with DS. The aim of this systematic review was to identify the podometric measurement tools used to typologically classify the footprints in the population with DS.

**Methods:**

The following electronic databases were searched for studies describing footprint measurement tools to assess and classify the foot types in patients with DS published from inception to December 2020: PubMed, Web of Science, CINAHL, and Scopus. Articles were initially searched by screening titles and abstracts. Potentially relevant studies were then further screened by reviewing full texts. Studies that met the inclusion criteria were included in the review.

**Results:**

Of the 122 articles identified by the search strategy, 14 full texts were retained to assess for eligibility, of which 11 studies met the inclusion criteria and were included. All the studies used footprint measurement methods to classify the foot types in subjects with DS, but only two studies assessed the reliability of those methods for the population with DS. The footprint measurement tools identified were a podoscope, a pressure-sensitive mat, a PressureStat^TM^ carbon paper, and a 3D scanner. The Arch Index was the most common footprint measurement analyzed (seven studies). Two studies used the “gold standard” indexes that include Hernández-Corvo Index, Chippaux-Smirak Index, Staheli Index, and Clarke Angle to measure footprints.

**Conclusions:**

There is a need to determine the reliability and validity of the footprint measurement methods used for clinical classification of the foot types in subjects with DS. This can contribute to an early diagnosis of foot abnormalities that would help to reduce mobility impairments, improving the quality of life of patients with DS.

## Background

Down syndrome (DS) is the most frequent chromosomal disorder, with an incidence of one in 660 live births [1]. Trisomy 21 is the cause of DS, and it affects multiple body systems including the nervous, cardiovascular, and musculoskeletal systems [2]. Individuals with DS have a variable degree of intellectual and physical disability [3]. Musculoskeletal abnormalities in DS, including hypotonia, ligamentous laxity, short extremities, and reduction of muscular strength, influence the subsequent development of misalignments of the lower limbs [4].

Normal foot morphology is essential for normal gait, and some foot disorders may be related to more proximal lower limb anomalies [5] that could interfere significantly with regular daily activities. In DS, the most prevalent foot variations are flat foot and pronated flat foot, calcaneal valgus, hallux valgus, and metatarsus primus varus [6]. Footprint evaluation is an important part of early podiatric medical diagnosis for detecting orthopedic problems of the lower limbs, especially the feet, in subjects with DS [7]. Two different approaches can be used to evaluate the morphological features of the foot: techniques to obtain footprints that require a manual assessment, which is usually tedious, and sophisticated instruments that immediately yield gold standard podometric indexes, but are more expensive and sometimes an expert is necessary to work with the equipments [6]. Instruments used to obtain footprints include ink imprints, optical podoscopes, baropodometry, pedography, digital photography, radiography, and platinum scanners, among others.

Footprint parameters are able to detect the main variations in foot morphology and provide relevant information for the management of orthopedic disorders [8]. Many studies have assessed the reliability and validity of different footprint measurement methods used for the clinical classification of foot types in the general population. However, studies should consider the specific morphotype and musculoskeletal variations of the foot in individuals with DS to identify accurate footprint instruments for this population. Therefore, the aim of this systematic review was to identify the podometric measurement tools which have been described in the scientific literature to assess and typologically classify the footprints in patients with DS.

## Methods

### Search strategy

This systematic review was conducted in accordance with the Preferred Reporting Items for Systematic reviews and Meta-Analyses (PRISMA) statement guidelines checklists and flow diagram [9]. A systematic search was conducted using the electronic databases PubMed, Web of Science, CINAHL, and Scopus, in December 2020. The search strategy used was ((down syndrome) AND (footprint OR foot print OR foot impression OR podometry OR podoscope OR baropodometry OR pedography OR podometric index OR arch index OR Hernandez-Corvo index OR Hernandez Corvo index OR Chippaux-Smirak index OR Chippaux Smirak index OR Staheli arch index OR Clarke angle OR foot types OR foot classification)).

No date restrictions were applied. Articles published in English and Spanish were included. Citation chaining was undertaken to identify any article that may have been missed in the search strategy.

### Study selection

Titles and abstracts of all articles were initially screened. If there was any doubt about the eligibility of an article, the full-text paper was retrieved. Potentially relevant studies were then further screened by reviewing full texts. Reference lists and citations of all retained studies were examined in an attempt to locate further studies. Studies were included if they met the inclusion criteria. The inclusion and exclusion criteria used for study selection are listed in Table 1.

**Table 1:** Inclusion and exclusion criteria

Inclusion criteria	Exclusion criteria
Studies using a podometric measurement tool for the assessment and classification of the footprints typologically	Articles that are literature reviews, short reports, letters, or congresses proceedings
Studies reporting that podometric measurement(s) were used to identify the foot types	Non-English nor Spanish publications
Studies based on both adults and children/adolescents with Down syndrome	Full-text article not available

### Data collection

The data extracted included sample characteristics (sample size, gender, age, participants with/without DS), study aims and hypothesis, and footprint methods (instrument used, measurements, protocol, foot type classification, and reliability and validity (if evaluated)).

## Results

**Fig. 1:**
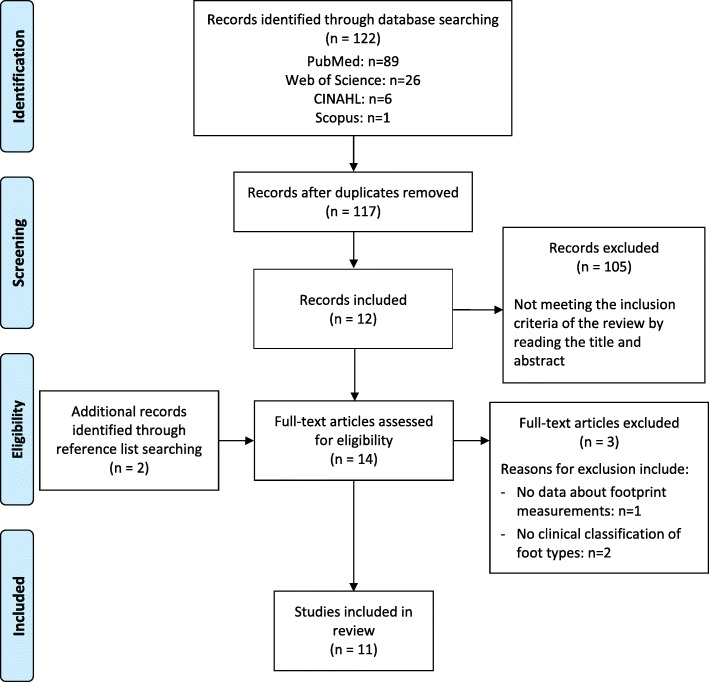
PRISMA flow diagram

A total of 122 articles were identified from the search strategy. After the removal of duplicates, the titles and abstracts of 117 studies were screened. Two additional studies were identified through searching of reference lists. Fourteen studies were retained for full-text screening and two of these were excluded for reasons listed in the PRISMA flow diagram (Fig. 1) [9]. Finally, 11 studies were included in the review.

### Characteristics and aims of included studies

The main characteristics of the 11 included studies are provided in Table 2. All studies assessed and typologically classified the footprints of participants, but the objectives of the studies were diverse. Five studies aimed to investigate the prevalence of foot deformities from footprint measurements in individuals with DS [5, 7, 12, 13, 16]. Two studies conducted by Galli et al. [10, 11] focused on the relationship between flat feet and the presence of gait alterations in children with DS. Other two studies from the same research group [15, 17] investigated the association between foot deformities and footwear-fitting problems in children and adolescents with DS. Finally, two studies evaluated the reliability of footprint measurement tools in a population with DS: Gutierrez-Vilahú et al. [6] study included adults and Hassan et al. [14] included children and adolescents.

**Table 2:** Characteristics of included studies, participants, podometric measurements, and foot type classification

Author, date	Sample size (***N***)	Females/males	Age [mean (SD) in years]	Participants	Study aim	Footprint instrument(s)	Measurements	Static/dynamic protocol	Footprint type classification
Concolino et∼al., 2006 [7]	C, 100 DS, 50	C, 68/32 DS, 31/19	C, 4.8 (range 3–8) DS, 5.6 (range 4–8)	C: children without DS DS: children with DS	To verify the importance of podiatric evaluation in patients with DS for early diagnosis and of minor orthopedic problems	Podoscope (polarized light) Electronic baropodometer (4800 active sensors over 120 cm)	N.S.	Both	Bony deformity of the forefoot, flat foot, isolated calcaneal valgus, knee valgus, and pronated flat foot
Galli et∼al., 2014 [10]	C, 15 DS, 29	N.S.	C, 9.2 (5.7) DS, 9.8 (2.3)	C: children without DS DS: children with DS	To quantitatively assess the relationship between the flat foot and the gait alterations in DS children	Pressure-sensitive mat (2016 sensing elements in a 42 × 48 matrix) Matlab® software	Arch Index	Static (barefoot, standing on the mat for 5s)	Flat foot
Galli et∼al., 2014 [11]	C, 15 DS, 55	N.S.	C, 9.2 (5.7) DS, 9.6 (1.7)	C: children without DS DS: children with DS	To determine if DS children with the flat foot are characterized by an accentuated external foot rotation during walking	Pressure-sensitive mat (2016 sensing elements in a 42 × 48 matrix) Matlab® software	Arch Index	Static (barefoot, standing on the mat for 5s)	Flat foot
Galli et∼al., 2016 [12]	HC, 19 HT, 13 HA, 14 DS, 13	N.S.	HC, 10.10 (1.93) HT, 13.96 (1.14) HA, 37.30 (7.42) DSC, 10.42 (2.46) DST, 17.20 (1.62) DSA: 27.90 (3.69)	HC, HT, HA: subjects without DS DS: subjects with DS (time range of 17 yr)	To quantify foot abnormalities in individuals with DS while standing, in terms of foot-ground interaction parameters from childhood to adulthood	Pressure-sensitive mat (2016 sensing elements in a 42 × 48 matrix) Matlab® software	Arch Index	Static (barefoot, standing on the mat for 5s)	Flat foot, contact pressure
Gutiérrez-Vilahú et∼al., 2015 [13]	C, 21 DS, 22	C, 10/11 DS, 11/11	C: M 20.45 (2.16); F 20 (1.70) DS: M 23.82 (3.12); F 24.82 (6.81)	C: subjects without DS DS: subjects with DS	To analyze, measure, and classify footprints on the basis of gold standard podiatric indices in young people with DS	Optical podoscope (chromed, direct 220 V light, 60 × 45 × 33) Digital camera	Hernández-Corvo Index Chippaux-Smirak Index Staheli Index Clarke Angle	Static (barefoot, standing, and lower limbs in parallel)	Flat foot, pronated foot, cavus foot
Gutiérrez-Vilahú et∼al., 2016 [6]	22	11/11	M 23.82 (3.12); F 24.82 (6.81)	Special school students with DS	To assess the reliability and validity of a footprint assessment technique in the population with DS	Optical podoscope (chromed, direct 220 V light, 60 × 45 × 33) Digital camera Photoshop CS5 software	Hernández-Corvo Index Chippaux-Smirak Index Staheli Index Clarke Angle	Static (barefoot, standing, and lower limbs in parallel)	N.S.
Hassan et∼al., 2020 [14]	30	18/12	10.6 (3.9)	Children and adolescents with DS	To determine the reproducibility of measuring foot dimensions of children and adolescents with DS using 3D scanning	FotoScan 3D scanner 3D-Tool© software version 13	Arch Index	Static (standing in a relaxed, full weight-bearing position)	Forefoot shape
Lim et∼al., 2015 [15]	50	28/14	10.6 (3.9)	Children and adolescents with DS	To determine if there is an association between flat feet, hallux valgus, and footwear-fitting problems with foot-specific disability in children and adolescents with DS	PressureStat^TM^ carbon paper Image analysis software Scion®	Arch Index	Static (standing relaxed)	Flat feet, hallux valgus, toe deformities
Mansour et∼al., 2017 [5]	C, 53 DS, 55	C, 26/27 DS, 19/36	C, 13.5 (11.2) DS, 14.6 (7.4)	C: asymptomatic subjects DS: patients with DS	To investigate the prevalence of foot deformities in patients with DS	Podoscope	N.S.	Static (standing)	Hallux valgus, syndactyly, grade II and grade III pes planus, joint laxity
Pau et∼al., 2012 [16]	C, 99 DS, 99	N.S.	C, 9.5 (1.6) DS, 9.7 (1.7)	C: children without DS DS: children with DS	To quantitatively characterize the main foot-ground contact parameters to assess foot evolution with age in young individuals with DS	Pressure-sensitive mat (2016 sensing elements in a 42 × 48 matrix) Matlab® software	Arch Index	Static (barefoot, standing on the mat for 5s)	Flat foot, contact pressure
Shields et∼al., 2017 [17]	50	22/28	10.6 (3.9)	Children and adolescents with DS	To investigate the relationship between foot structure, footwear fit, and levels of physical activity in children and adolescents with DS	PressureStat^TM^ carbon paper Image analysis software Scion®	Arch Index	Static (standing relaxed)	Flat foot, footwear

*Abbreviations*: *C*, control; *DS*, Down syndrome; *DSC*, Down syndrome children; *DST*, Down syndrome teenagers; *DSA*, Down syndrome adults; *F*, females; *HC*, healthy controls; *HT*, healthy teenagers; *HA*, healthy adults; *N*, number of participants; *N.S.*, not stated; *M*, males; *SD*, standard deviation

### Population

The samples of all included studies were based on subjects with DS, and seven studies [5, 7, 10–13, 16] also included a control group consisting of individuals without DS. Regarding the age of participants, the studies included children [7, 10, 11, 16], children and adolescents [5, 14, 15, 17], or young adults [6, 12, 13]. Participants were recruited from a rehabilitation center [10–12, 16], a special school [6, 13], or a community-based organization for individuals with DS [14, 15, 17]. The remaining two studies [5, 7] did not state the recruitment site of the participants.

### Footprint measurement instruments

All the included studies collected footprint images to identify the foot types of participants. The footprint measurement instruments used differ between studies. Concolino et al. [7] used a podoscope with polarized light, and the collected podiatric data were subsequently compared and expanded using an electronic baropodometer, composed of a platform with 4800 active sensors over 120 cm and a walking distance measuring 220 cm. An optical podoscope with direct 220 V light and a digital camera were used to photograph the footprints of participants in two studies conducted by the same research group [6, 13]. Other groups used a pressure-sensitive mat, composed of 2016 sensing elements arranged in a 42 × 48 matrix and connected to a personal computer to register plantar pressure measurements in four studies [10–12, 16]. To identify foot type, the participants were placed on the mat with the help of an assistant who asked them to stand as still as possible for 5-s trials. Two studies [15, 17] obtained the footprint using the PressureStat^TM^ carbon paper with the participant standing relaxed. Finally, Hassan et al. [14] used the FotoScan 3D scanner that consists of a fixed system of cameras and projectors to obtain images of the foot, which are automatically converted into a 3D model.

### Footprint measurements

Five footprint measurements were identified. These were Arch Index (AI) and the four indices included in the reference “gold standard,” which are Hernández-Corvo Index (HCI), Chippaux-Smirak Index (CSI), Staheli Index (SI), and Clarke Angle (CA). AI is the ratio of the area of the middle third of the footprint to the entire footprint area and gives an indicator of arch height [18]. The gold standard indices serve to calculate the surface contact of the footprint [19].

AI was the most common footprint measurement, as it was analyzed in seven studies [10–12, 14–17]. In the two studies conducted by Gutiérrez-Vilahú et al. [6, 13], the photographic foot images were used to calculate the podiatric indices HCI, CSI, SI, and CA (gold standard). Two studies [5, 7] did not report the measurement used to classify the foot types of participants.

### Static or dynamic protocol

According to the protocols described, 10 of the included studies measured the static footprints with the participant standing barefoot. Only one study [7] used a complete podiatric examination: clinical orthopedic observation, podoscope appraisal, and static and dynamic baropodometric examination analyzed both static and dynamic footprints collected during walking.

Of the 11 included studies, 9 collected footprint images from both feet, one study [14] only scanned the footprint of the right foot of participants, and another study [5] did not state whether one or both feet were analyzed.

### Reliability of footprint measurement methods

Gutiérrez-Vilahú et al. [6] assessed the reliability and validity of the use of Photoshop CS5 software, previously validated in the general population, to measure footprints in young adults with DS. The reliability test for the Photoshop CS5 method showed very good values of the intraclass correlation coefficient (ICC) for all of the indices, ranged from 0.984 for the HCI to 0.995 for the CA. Validity testing also found very good ICC values, which were equal to or greater than 0.988 for all the podiatric indices [6].

Hassan et al. [14] determined the reproducibility of measuring foot dimensions of children and adolescents with Down syndrome using 3D foot scanning. The intra-rater reproducibility (ICC ranged from 0.74 to 0.99) and inter-rater reproducibility (ICC ranged from 0.73 to 0.99) values indicated moderate to excellent reliability for all foot dimension measurements [14].

### Footprint type classification

Persons with DS showed several orthopedic anomalies. In relation to the results of the study, Concolino et al. [7] showed bony deformity of the forefoot, flat foot, isolated calcaneal valgus, knee valgus, and pronated flat foot. Mansour et al. [5] determined foot deformities were found in the DS group: hallux valgus, syndactyly between the 2nd and 3rd toes, grade II pes planus and grade III pes planus, and joint laxity and the presence of an increased space between the 1st and 2nd toes. Gutiérrez-Vilahú et al. [13] classified flat foot and/or pronated foot according to HCI, CS, SI, and CA. In healthy subjects, the cavus foot was determined according to CSI and SI, while a normal foot based on CA was shown. Several studies of the AI determine flat foot. In Galli et al. [10], the AI data showed lower values for flat feet. Furthermore, ankle plantar flexion moment and ankle power during terminal posture were significant in differentiating patients with and without flat feet.

Data obtained in Galli et al. [11] demonstrate that throughout the gait cycle, children with DS and flat feet were characterized by greater additional foot rotation compared to those without flat feet and controls. In Galli et al. [12] and Pau et al. [16] in terms of contact pressures and flat feet, changes were found in healthy individuals in adolescence and adulthood for all regions of the foot. Individuals with DS present with hypotonia and ligamentous laxity, which is why they observed significant increases in the forefoot and hindfoot only in adulthood.

Hassan et al. [14] described some measurements forefoot, rearfoot in relation to foot length. Lim et al. [15] relate the use of footwear to the AI, flat feet, hallux valgus, and lesser toe deformities.

Only a study by Shields et al. [17] relates flat feet and footwear fit which was negatively associated with an activity.

## Discussion

The objective of this systematic review was to identify the podometric measurement instruments used to assess and typologically classify the footprints in patients with DS. Eleven studies were included in the review and, of these, only two studies assessed the reliability of footprint measurement tools in the population with DS.

It is widely recognized that subjects with DS often show marked alterations in the structure and functionality of the foot that, along with other typical DS features, are responsible for pain, postural, and gait disturbances [1, 3, 7, 12, 20–24]. One of the most common abnormalities is the flat foot, which is present in 60% of individuals with DS [7, 16]. Other common foot alterations in DS include calcaneal valgus, hallux valgus, and metatarsus primus varus, all of which can be diagnosed by podometric measurements of footprints. Since these abnormalities can significantly interfere with the normal daily activities of these patients, it is important to carefully monitor foot development in children and adolescents with DS, to reduce the risk of mobility problems and minimize the possible consequences in adulthood [25].

In this systematic review, all the included studies used footprint measurement methods to assess and typologically classify the footprints of patients with DS, but with different purposes. Five studies investigated the prevalence of foot deformities in individuals with DS, two studies focused on the relationship between flat feet and the presence of gait alterations in children with DS, and two studies examined the association between foot deformities and footwear-fitting problems in children and adolescents with DS. The footprint measurement methods used in the studies include a podoscope (four studies), a pressure-sensitive mat (four studies), a PressureStat^TM^ carbon paper (two studies), and a 3D scanner (one study).

Regarding footprint measurement, the AI and the podiatric indices included in the gold standard (HCI, CSI, SI, and CA) were calculated in seven and two studies, respectively, for the classification of foot types from the footprint images. These footprint parameters use different classification criteria to identify the distribution of different foot types. Nikolaidou and Boudolos [8] examined the simultaneous use of the parameters AI, CSI, K Index (KI), and Footprint Angle (FPA) in the classification process (co-classification) in an attempt to provide a rational classification of foot types in young school children. They found that AI had the lowest percentage of misclassified cases, suggesting its strong classifying ability in the co-classification process. In contrast, the ability of CSI, KI, and FPA in classifying foot types when other footprint parameters are involved in the classification process seemed limited [8]. These authors considered that AI gave the lowest percentages of misclassified cases during the co-classification process. The co-classification model with the 4-cluster solution is proposed, and confidence limits are reported for a rational classification of feet in young school children.

Finally, we found only two studies that assessed the reliability of a foot measurement method. The studies conducted by Gutiérrez-Vilahú et al. [6] and Hassan et al. [14] evaluated the use of the Photoshop CS5 software and the FotoScan 3D scanner, respectively, to measure footprints in subjects with DS. In the first case, the authors concluded that the computerized measurement technique analyzed was reliable and valid for obtaining the gold standard podometric indices (the HCI, CSI, AI, and CA) of the footprint in the DS population. The second study found that the measurement of specific foot dimensions of children and adolescents with DS using 3D scans was reproducible. Therefore, by using these methods, clinicians can perform measurements of foot dimensions to monitor the foot shape of children with DS, or to provide an appropriate management of foot abnormalities in subjects with DS. As far as we know, all the previous methods have been validated in the general population. Specifically, for Down syndrome, the Photoshop CS5 has also been validated for the Down syndrome population, but Hassan et al. only showed the reproducibility of their method in this population. It is recommended that the measurement methods be previously reliable and validated in the general population, because it is also necessary to make reliable and validate these same measurement methods in special populations such as DS.

## Conclusions

In Down syndrome, foot abnormalities, including flat foot, pronated flat foot, calcaneal valgus, hallux valgus, and metatarsus primus varus, are common. A wide variety of footprint measurement methods to evaluate and clinically classify the foot types have been validated for the general population. Conversely, the results from this review indicate that very few studies have assessed the reliability of these footprint measurements in individuals with DS. For this reason, there is a great need to validate the footprint measurement methods specifically in the population with DS, as it can contribute to an early diagnosis of foot abnormalities that helps reduce mobility impairments, improving the quality of life of patients with DS.

## Data Availability

Not applicable

## Abbreviations

AI: Arch Index
CA: Clarke Angle
CSI: Chippaux-Smirak Index
DS: Down syndrome
FPA: Footprint Angle
HCI: Hernández-Corvo Index
ICC: Intraclass correlation coefficient
KI: K Index
PRISMA: Preferred Reporting Items for Systematic reviews and Meta-Analyses
SI: Staheli Index
